# Influence of Inhibitors Generated in Lignocellulosic Hydrolysates from Group of Acids on the Growth of Strains TG1 and Tuner of *Escherichia coli*

**DOI:** 10.3390/microorganisms13030605

**Published:** 2025-03-05

**Authors:** Suelen S. Gaspar, Júnia Alves-Ferreira, Patrícia Moniz, Talita Silva-Fernandes, Adriana I. R. Silvestre, Ivone Torrado, Gaetano R. Pesce, Florbela Carvalheiro, Luís C. Duarte, Maria C. Fernandes

**Affiliations:** 1 Alentejo Biotechnology Center for Agriculture and Agro-Food (CEBAL)/Polytechnic Institute of Beja (IPBeja), Apartado 6158, 7801-908 Beja, Portugal; suelen.gaspar@cebal.pt (S.S.G.); junia.caturra@ipbeja.pt (J.A.-F.); patricia.moniz@cebal.pt (P.M.); talita.fernandes@cebal.pt (T.S.-F.); ivone.torrado@ipbeja.pt (I.T.); 2 MED—Mediterranean Institute for Agriculture, Environment and Development & CHANGE—Global Change and Sustainability Institute, CEBAL-Alentejo Biotechnology Center for Agriculture and Agro-Food, Apartado 6158, 7801-908 Beja, Portugal; 3 Forest Research Centre, TERRA Associate Laboratory, School of Agriculture, University of Lisbon, Tapada da Ajuda, 1349-017 Lisbon, Portugal; 4 LNEG—National Laboratory of Energy and Geology, Bioenergy and Biorefineries Unit, Estrada do Paço do Lumiar, 22, 1649-038 Lisbon, Portugal; florbela.carvalheiro@lneg.pt (F.C.); luis.duarte@lneg.pt (L.C.D.); 5 Department of Agronomy, Food, Natural Resources, Animals and Environment—DAFNAE, University of Padua, Agripolis Campus, Viale dell’Università 16, 35020 Legnaro, PD, Italy; gaetano.pesce@unipd.it

**Keywords:** biomass, bioproducts, fermentation, microorganisms

## Abstract

Concerns over fossil fuels are of increasing interest in biorefineries that utilize lignocellulosic residues. Besides sugars, inhibitors are formed during biomass pretreatment, including acetic acid (AI) and formic acid (FI), which can hinder microbial fermentation. The TG1 and Tuner strains of *Escherichia coli* were subjected to various acid concentrations. Samples were taken during fermentation to monitor growth, sugar consumption, biomass yield, and product yield. With increasing AI, the TG1 strain maintained stable growth (0.102 1/h), while xylose consumption decreased, and product formation improved, making it better suited for high-acetic-acid industrial applications. In contrast, the Tuner strain performed better under low-inhibitor conditions but suffered metabolic inhibition at high AI levels, compensating by increasing lactic acid production—an adaptation absent in TG1. However, Tuner showed greater resistance to formic acid stress, sustaining higher growth and ethanol production, whereas TG1 experienced a greater metabolic decline but maintained stable acetic acid output. Both strains experienced inhibition in formic acid metabolism, but TG1 had a higher yield despite its lower overall robustness in formic acid conditions. The use of TG1 for value-added compounds such as ethanol or formic acid may help to avoid the use of chemicals that eliminate acetic acid. Tuner could be used for lactic acid production, especially in hydrolysates with under moderate concentration.

## 1. Introduction

In the scope of sustainability, it is imperative to produce bioproducts from renewable sources and environmentally appropriate resources. Currently, there is growing worldwide interest in the concept of biorefineries aiming at the sustainable production of a wide variety of biofuels and more valuable bioproducts such as precursors or products for the chemical, food, textile, feed, and pharmaceutical industries [1,2]. Concerns about the exhaustion of the global reserves of fossil fuels and, mainly, the environmental concerns associated with fossil fuels have driven the search for renewable energy sources with high added value and with significant agricultural and industrial importance.

Lignocellulosic biomass (LCB) obtained from a wide range of sources (agricultural, forestry, industrial, and anthropogenic uses) exceeds the global energy needs [3]. According to Brosowski [4], Germany uses between 66% and 84% of its biomass potential, indicating that a part has still not been exploited. Lignocellulosic biomass is considered a potential raw material in biorefineries due to its low cost, high availability, and high content of fermentable sugars with high potential for bioconversion into value-added products for specific industries [5,6]. However, lignocellulosic materials are recalcitrant, which means they have a complex molecular structure, formed mainly by three different polysaccharide fractions—cellulose, hemicellulose, and lignin—whose recovery can be facilitated through selective fractionation [7,8,9,10,11]. For the sugars present in the lignocellulosic biomass to be used, namely pentoses and hexoses [12], the biomass must be previously subjected to a physical, chemical, mechanical pretreatment, or a combination of these [13], which will improve the yield of the subsequent stage of fermentation [14,15,16,17,18].

Unfortunately, pre-treatment releases sugars and compounds that inhibit microbial metabolism, hindering bioprocess performance. These must be removed or reduced, requiring costly detoxification methods. These compounds are classified into the following three main groups: furan derivatives, phenolic compounds, and aliphatic organic acids. Among the acid group inhibitors, acetic acid, released by the deacetylation of hemicelluloses, is the most abundant in hydrolysate. Formic and levulinic acids are products of sugar degradation [19,20]; although they are present in lower concentrations, formic acid can be more toxic due to the high permeability of the membrane [19,21,22,23].

In this context, it has been recognized that the implementation of the biochemical platform within the scope of the biorefinery concept is dependent on the improvement in the pretreatment technology. The inhibitory effect is considered the main bottleneck for the generation of bioproducts from lignocellulosic materials in biorefineries [24,25]. Therefore, it is essential to understand the impacts of these compounds to enhance the fermentative performance. Regardless of the methods and pretreatment conditions for the lignocellulosic biomass, the formation of inhibitors that represent a threat to the microorganisms present during the fermentation stage appears to be a general phenomenon [26,27] and, consequently, the mitigation of these toxic substances is a necessary step towards efficient fermentation. Instead of ethanologenic microorganisms that are widely used in industry, such as *Saccharomyces cerevisiae*, enteric bacteria like *Escherichia coli* can metabolize a wide range of sugars, hexoses, and pentoses, including glucose and xylose, present in hemicellulosic hydrolysates [28,29,30] and have a high tolerance to several toxic inhibitors compounds [25,31,32,33]. *E. coli* is one of the most used biocatalysts in biorefineries to obtain bioproducts, such as second-generation ethanol, D-lactic acid, succinic acid, and 1,4-butanediol [34,35].

Although recent works have presented the mechanisms associated with acid stress in *E. coli* [26,27,35], studies of biorefinery factory cells, such as *E. coli*, related to inhibitor tolerance are very scarce [36] and have used very different cultivation conditions, which make their comparative assessment difficult. Therefore, through a comparative study (benchmarking), this work aims to analyze the growth kinetics of different strains of *E. coli*, namely TG1 and Tuner, in different concentrations of inhibitors of the group of acids (acetic and formic) generally present in lignocellulosic hydrolysates to analyze their tolerance to these inhibitors and determine the viability of using these strains in a biorefinery. The yields of biomass and products were obtained through stoichiometric calculations. These initial results will be used to determine if there is a resistance mechanism that can later be applied to another cell.

## 2. Materials and Methods

### 2.1. Microorganisms and Inocula Preparation

The TG1 [ *supE thi-1*Δ *(lac-proAB)* Δ *(mcrB-hsdSM)5 (r_K_ ^−^ mK ^−^) F′ traD36 proAB lac^q^Z* Δ *M15*] and Tuner strains (F^−^ *ompT hsdS*_B_ (r_B_^−^ m_B_^−^) *gal dcm lacY1*(DE3) were used as model cell factories. Both are commercial strains available for molecular biology studies and were supplied by GE Amersham Pharmacia Biotech (Amersham, UK) and Novagen (Darmstadt, Germany), respectively.

Stock cultures of these strains were prepared by growing each of them in Luria Bertani medium and then stored in cryovials at −80 °C in the presence of glycerol (20%). The cultivations were performed in 1 L baffled Erlenmeyers at 200 rpm and 37 °C, and the cells were collected at the end of the exponential phase, after approximately 9 h of cultivation.

### 2.2. Preparation of SelecTEcoli’s Mineral Media (SMM)

For the cultivation and growth of *E. coli,* an extensive bibliographic search was carried out on liquid media. The media M9 [36], AM1 [37], NBS [37], YNB [38], and MB [39] were used as a basis for formulating a chemically defined liquid media, specifically formulated for this work, to try to better simulate the lignocellulosic hydrolysate using xylose (HIMEDIA; Thane, India) as a carbon source. The composition of the medium used, called SMM (SelecTEcoli Mineral Medium), is shown in Table 1.

The solutions of vitamins, trace elements, inhibitors, and betaine were individually sterilized using membrane filters of 0.22 µm (Gelman Sciences, Ann Arbor, MI, USA, EUA), and the remaining solutions were sterilized in an autoclave (121 °C, 15 min). Trace element and vitamin solutions were used in the SMM media at 2.0 mL/L and 1.0 mL/L, respectively.

### 2.3. Fermentation

The prepared inocula of *E. coli* strains were slowly thawed and centrifuged at 20,000× *g*. The pellet was resuspended in the SMM, reaching an initial OD (600 nm) of approximately 0.2. The fermentation was conducted in 400 mL mini reactors, as reported in Alves-Ferreira et al. [40], with a working volume of 200 mL. Components of the media were added to each reactor, and different concentrations of inhibitors were added as follows: acetic acid (AI): 8, 16, and 20 g/L; formic acid (FI): 1.5, 3, and 6 g/L. For the initial pH adjustment to 7.0, KOH 4 M was added. During the fermentation assay, the pH was adjusted automatically by KOH (2 M) addition.

The experiments were conducted at least in duplicate, under agitation of 250 rpm, at 37 °C for 72 h. Samples (2 mL) were aseptically collected with the aid of sterile syringes attached to the reactors at 0, 3, 6, 9, 24, 32, 48, 56, and 72 h. The pH values and consumed base volume were monitored continuously [41]. Cell concentration was measured at 600 nm using a double beam spectrophotometer (Helios Alfa, Thermo Scientific, Hercules, CA, USA) [31,42,43] and then centrifuged at 12,000 rpm for 5 min at 4 °C; the supernatant was stored for further analysis. The sugar composition and fermentation product formation were measured by HPLC (see below).

### 2.4. Analysis

After thawing, the supernatants were filtered through 0.22 µm membrane filters and analyzed by HPLC using a Thermo Scientific system (Hercules, CA, USA) equipped with a refractive index detector (Refractomax521) controlled at 39 °C and a diode array detector (DAD 3000). Briefly, the sugars (monosaccharides) and organic acids were analyzed using a Bio-Rad Aminex HPX-87H column (300 × 7.8 mm) (Hercules, CA, USA) at 50 °C, with a 5 mM H_2_SO_4_ aqueous solution as the mobile phase and a flow of 0.6 mL/min. Detection was performed as described in [44]. The R^2^ of the standards curves used in the present work were higher than 0.999809.

### 2.5. Calculations

The reported growth kinetic data are based on the OD/absorbance measurements and the logarithm linearization of the exponential growth equation, as follows:

Ln (Ab_t_/Ab_0_) = µ × t (1)

where Ab_t_ and Ab_0_ are OD (600 nm) at time t and the initial OD (600 nm), respectively, and µ is the specific growth rate. The linear regression calculations were carried out using MS Excel. The lag phase duration (λ, h) was estimated as the intercept of this equation with xx axis (time axis).

The xylose consumption rate (*Q_xyl_*) was obtained by Equation (2).

(2) $$Q_{Xyl}=\frac{{\left[Xyl\right]}_{t}-{\left[Xyl\right]}_{0}}{t}$$

where [*Xyl*]*_t_* corresponds to the measured xylose concentration (g/L) at time *t* (h), and [*Xyl*]*_0_* corresponds to the initial xylose concentration.

The relative xylose consumption was calculated using the Equation (3).

(3) $$Xylose\;consumption(\%)=\frac{{\left[Xyl\right]}_{0}-[{Xyl]}_{t}}{{[X}_{yl}{]}_{0}}\times 100$$

Similar equations were used to estimate the inhibitors’ consumption, when relevant.

The biomass yield was calculated as the ratio of the dry cell weight (DCW) produced in a given time interval to the xylose consumption (*Q_Xyl_*) in the same interval. The DCW data were estimated from the OD (600 nm) data using the following relation: 1 OD (600 nm) = 0.37 g_DCW_/L [43].

Product yield (Y_P/S_ was calculated according to Equation (4) [41].

(4) YPS=grams of generated product grams of consumed substrate=[P]f−[P]0[Xyl]0−[Xyl]t

where [*P*]*_f_* is the final concentration of each product, and [*P*]_0_ is the initial concentration of each product (usually zero).

### 2.6. Statistical Analysis

The specific growth rate, Q_xyl_, xylose consumption (%), Y_X/S_, and all the Y_P/S_ were subjected to an analysis of variance (ANOVA). The means for each trait were separated using Fisher’s least significant difference test, with a significance threshold of 0.05. Two-way ANOVAs were performed, considering the two strains (S) and four acid concentrations (A) (S × A). Subsequently, only the significant interactions were analyzed; in the absence of these, the main effects were examined. Values expressed as percentages underwent angular transformation prior to the analysis of variance.

## 3. Results and Discussion

In the present work, the performance and tolerance of the *E. coli* strains, TG1 and Tuner, were evaluated in SMM media supplemented with 20 g/L of xylose, in the presence of different concentrations of the inhibitors of acetic acid and formic acid, which were generated during the process of pretreatment of the lignocellulosic biomass.

Although there are several genetically modified strains, TG1 and Tuner were chosen so that we could understand their mechanism in the presence of inhibitors and analyze their use in biorefineries. The TG1 strain was selected because it was previously proven to be a robust strain with xylose-efficient assimilation, while the Tuner strain was chosen for its promising fermentative performance. The microorganisms that are able to ferment lignocellulosic hydrolysates require nutrients [45]. The SMM media contains a significant amount of nutrients, such as proteins, vitamins, and minerals, and was developed specifically to favor the growth of *E*. *coli* strains since the literature suggests that supplementing the media with complex nutrient sources can increase the yield and productivity of fermentations [46]. This study on the effects of acetic and formic acid inhibitors on *E. coli* strains TG1 and Tuner has significant implications for industrial biotechnology, particularly in biofuel and biochemical production. Understanding how these strains respond to the inhibitory compounds commonly found in lignocellulosic hydrolysates provides valuable insights for improving fermentation efficiency while making bio-based processes more viable.

### 3.1. Growth Evaluation

#### 3.1.1. TG1 Strain

**Figure 1:**
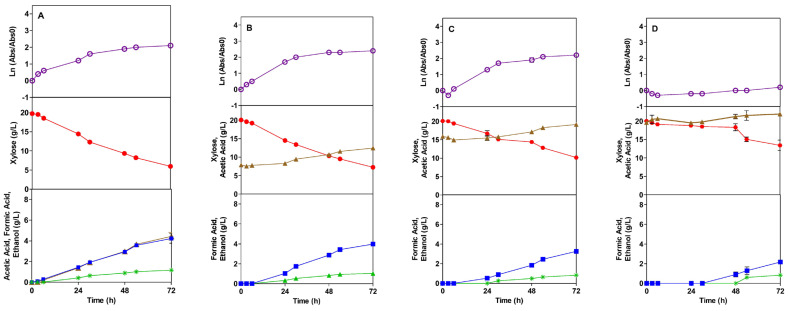
Fermentation profile of *E. coli* TG1 in SMM media in the absence (control) (**A**) and in the presence of the acetic acid inhibitor (AI) at concentrations of 8.0 (**B**), 16.0 (**C**), and 20.0 (**D**) g/L: growth kinetics, xylose consumption, and formation of products during 72 h of the fermentation process, respectively. Legend: Ln (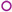), Xylose (●), Acetic Acid (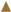), Formic Acid (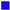), Ethanol (*).

**Figure 2:**
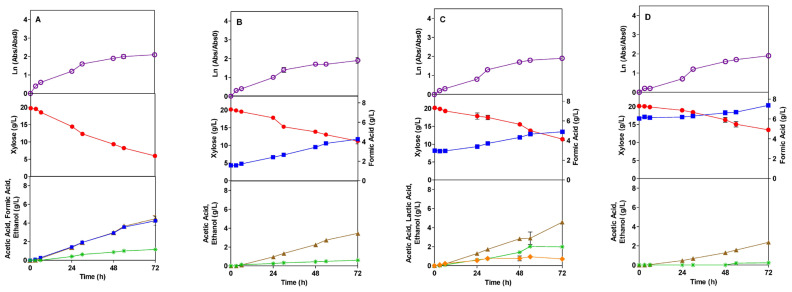
Fermentation profile of *E. coli* TG1 in SMM media in the absence (control) (**A**) and in the presence of the formic acid inhibitor (FI) at concentrations of 1.5 (**B**), 3.0 (**C**), and 6.0 (**D**) g/L: growth kinetics, xylose consumption, and formation of products during 72 h of the fermentation process, respectively. Legend: Ln (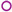), Xylose (●), Acetic Acid (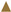), Formic Acid (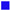), Ethanol (*), Lactic Acid (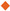).

The TG1 strain fermentation profile in the presence of the acetic acid inhibitor (AI) and the formic acid inhibitor (FI), are shown in Figure 1 and Figure 2, respectively. In the control assay, a diauxic phenomenon is observed right at the beginning of the fermentation, between 0 and 9, and after 24 h. The diauxic profile could be explained by glycerol consumption, probably resulting from the inoculum, and where the bacteria consume very little xylose. In the presence of the acetic acid inhibitor (AI), at 8 and 16 g/L, the growth curve does not present the diauxic behavior as the control, achieving a slightly higher absorbance than the control assay at the end of the fermentation. This can be explained by greater carbon source availability (xylose and AI). Higher concentrations of AI, 16.0 and 20.0 g/L, present lag phases of 4.7 and 44.6 h, respectively. With 20 g/L, the TG1 strain has a very long lag phase; after that, growth is very slow until the end of the experiment, presenting practically no positive ratio compared to the initial inoculation. During the exponential growth period, the obtained specific growth rate is 0.102 1/h for the control assay and 0.099, 0.104, and 0.011 1/h in the presence of 8, 16, and 20 g/L of AI, respectively (Table 2). Although, for 8 g/L, the specific growth rate value is slightly lower than the control, only with 20 g/L does the TG1 strain have a statistically lower value. The statistical analysis of the specific growth obtained shows that acetic acid concentrations up to 16.0 g/L do not affect the bacteria growth of this strain, whereas 20.0 g/L leads to a reduction in growth rate by 89% (Table 2), as compared to the TG1 control assay.

In the presence of the formic acid inhibitor (FI), TG1 presented a growth decrease when the FI concentration increased. Conversely, there was no lag phase in AI, which could be explained by the lower concentration of FI studies compared to AI studies. The values chosen for the present work are based on average values of both AI and FI, which are usually present in lignocellulosic biomass hydrolysate. Compared to the control assay, the presence of FI at concentrations of 1.5, 3.0, and 6.0 g/L reduced the specific growth rate of the TG1 strain by 40, 52, and 67%, respectively. These results show that formic acid has a higher impact on growth at lower concentrations than acetic acid.

#### 3.1.2. Tuner Strain

**Figure 3:**
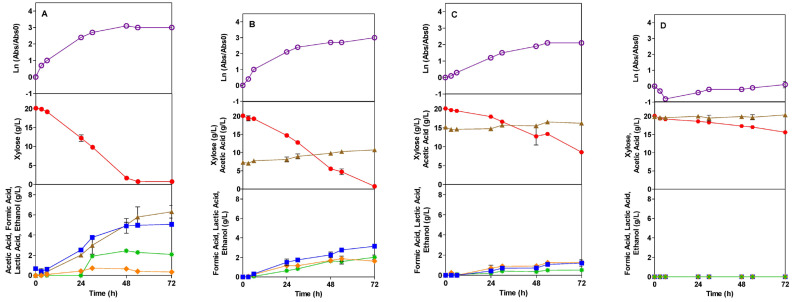
Fermentation profile of *E. coli* Tuner in SMM media in the absence (control) (**A**) and in the presence of the acetic acid inhibitor (AI) at concentrations of 8.0 (**B**), 16.0 (**C**), and 20.0 (**D**) g/L: growth kinetics, xylose consumption, and formation of products during 72 h of the fermentation process, respectively. Legend: Ln (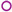), Xylose (●), Acetic Acid (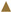), Formic Acid (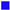), Ethanol (*), Lactic Acid (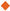).

**Figure 4:**
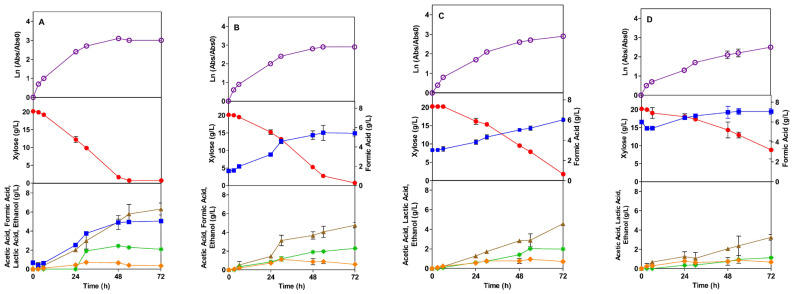
Fermentation profile of *E. coli* Tuner in SMM media in the absence (control) (**A**) and in the presence of the formic acid inhibitor (FI) at concentrations of 1.5 (**B**), 3.0 (**C**), and 6.0 (**D**) g/L: growth kinetics, xylose consumption, and formation of products during 72 h of the fermentation process, respectively. Legend: Ln (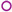), Xylose (●), Acetic Acid (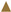), Formic Acid (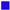), Ethanol (*), Lactic Acid (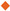).

The Tuner strain fermentation profile in the presence of the acetic acid inhibitor (AI) and the formic acid inhibitor (FI) are shown in Figure 3 and Figure 4, respectively. In the absence of an inhibitor, this strain has a shorter and higher cell growth profile than the TG1 strain, since the exponential growth occurs between 0 and 6 h. The specific growth rate for the control assay is 0.174 1/h, (Table 2), higher than the rate obtained for the TG1 strain.

In the presence of AI, the growth profile is more affected as the AI concentration increases, being practically killed in the presence of 20 g/L of acetic acid. In fact, for 16 and 20 g/L of AI, Tuner presented a lag phase of 0.4 and 64.9 h. With 20 g/L, only at the end of fermentation does the strain match the initial absorbance, showing a deeper decrease in the first few hours when compared to the TG1 strain. The obtained growth rates are 0.16, 0.051 and 0.013 1/h for 8, 16, and 20 g/L of AI, respectively (Table 2). As shown at 8 g/L, there is no difference with the control assay, but at concentrations of 16 and 20 g/L, the specific growth rate is significantly affected, showing a reduction in its values to 71% and 92%, respectively, of the control assay (Table 2). In the presence of FI, the Tuner strain also exhibits a similar behavior as observed in the presence of AI. Yet no lag phase was observed. Compared to the control assay, the presence of FI at concentrations of 1.5, 3.0, and 6.0 g/L reduced the exponential growth rate of the strain by 18, 23, and 34%, respectively.

Comparatively, in the presence of FI, the TG1 strain was more affected and underwent larger decreases in cell growth compared to Tuner, indicating that Tuner is more resistant to formic acid than TG1. The effects of strong interactions between the “Strain” factor and the “AI” were observed on the specific growth rate (Table 2). Indeed, while the specific growth rate for Tuner decreased with the increasing AI concentration (from 0.174 1/h of the control to 0.013 1/h at 20 g/L), for TG1 it remained stable for the control, at 8 g/L, and at 16 g/L (on average 0.103 1/h), before dropping sharply at 20 g/L (−87%) (Table 2).

Remarkably, both strains had to spend a great quantity of energy to maintain homeostasis and remain active during the fermentation process in the presence of high concentrations of AI (16.0 and 20.0 g/L). With a pH of 7.0 maintained throughout the fermentation process, acetic acid (pKa 4.8) was mainly dissociated in the form of acetate [47], allowing the studied strains to endure such high concentrations of the acid in the growth media. Yet this same fact allowed the passage of the acid’s protonated form into the cell, decreasing its pH and demanding energy to maintain the cell homeostasis [48]. Also, the presence of acetate in the intermembrane space probably increased cell turgor [49]. To overcome this situation, adaptations in cell physiology to cope with accumulated acetate anions may contribute to a reduced cell growth rate, especially at higher concentrations. Acetate is the most studied organic acid inhibitor in *E. coli* and is a natural fermentation product known for accumulating due to overflow metabolism and inhibiting cell growth, as well as acting as preservatives in food products [23,50]. Acetate levels depend on the type of cellulosic biomass and the pretreatment method. Furthermore, it has been shown that organic acids mainly inhibit cell mass production but not fermentation itself, as the fermentation process persists [47].

### 3.2. Sugar Consumption

The profile of xylose consumption is shown in Figure 1, Figure 2, Figure 3 and Figure 4, in the presence of AI and FI, respectively. The *E. coli* bacteria can grow in mineral media, as well as in hydrolysates of agro-industrial residues, using glucose and xylose as carbon sources under different fermentation conditions [51,52]. In this context, xylose was chosen as a carbon source to be used in this work. High amounts of pentose are known to have an inhibitory effect on the growth of *E. coli* [53]; therefore, the amount of 20.0 g/L of xylose was used.

Considering the total of 72 h of the fermentation process in the control experiment, 30% and 4% of the xylose was not metabolized by strains TG1 and Tuner, respectively.

The effects of strong interactions between the “Strain” factor and the “AI” were observed in the percentage of xylose consumption and Q_xyl_ (Table 2). By examining the significance letters, it is evident that the percentage of xylose consumption by the two strains in response to the AI exhibited a similar pattern to that of Q_xyl_ (Table 2). Specifically, Tuner’s values declined at concentrations above 8 g/L (−40% from 8 to 16 g/L and −61% from 16 to 20 g/L), while TG1 was less sensitive to the increasing concentrations (Table 2). However, xylose consumption (%) and Q_xyl_ were higher for the Tuner strain compared to TG1. Overall, the higher the Q_xyl_, the greater the quantity of cells produced.

The effects of significant interactions (*p* < 0.01) between the “Strain” and “FI” factors were observed on the percentage of xylose consumption and Q_xyl_ (Table 3). Indeed, the values for Tuner did not change significantly from 0 to 3 g/L, with an average of 94.6% xylose consumption and a mean Q_xyl_ of 0.27 g/L h. However, at 6.0 g/L of FI, xylose consumption dropped to 56.3%, while Q_xyl_ decreased to 0.16 g/L h (Table 3). Regarding TG1, the percentage of xylose consumption across the three FI concentrations did not show significant differences, with very similar values at 1.5 and 3.0 g/L of FI (44% and 43%, respectively) (Table 3). A similar pattern was observed for Q_xyl_, where the values at 1.5 and 3.0 g/L of FI equal 0.12 g/L h (Table 3).

The TG1 and Tuner strains are not selective for a single sugar, since both strains give preference to the glycerol present at the beginning of the fermentation process, and only after glycerol ends, at 6/9 h, does the bacteria fully consume the xylose monosaccharide. The xylose present in the media was not consumed in its entirety by any of the strains, as was also reported by [54]. In contrast, Utrilla et al. [42], using a different strain of *E. coli* (JU15) to produce lactic acid, observed that the strain consumed all the sugars present in the hydrolysate. The low amounts of inhibitors did not affect the absorption of sugars or metabolic activity of cells, as was also observed in other studies [55,56]. Although the Tuner strain consumes xylose faster and better than TG1, the TG1 strain is more affected by the presence of FI, while the Tuner strain by the presence of AI.

### 3.3. Biomass Yield

The development of each of the fermentations, the biomass yields (Y_X/S_), and the yields of the products (Y_P/S_) are presented in Table 2 and Table 3.

The TG1 biomass yield was 0.031 g/L, while the Tuner strain yield was twice that of TG1 (0.071 g/L). In the presence of AI, the biomass yield of the TG1 strain with 8.0 and 16.0 g/L of AI reached yield rates of 0.007 and 0.013 higher than those found in the control experiment, except for the AI concentration of 20.0 g/L, where the biomass yield was severely affected by the higher concentration of AI and resulted in a decrease of 0.029 in the yield. Conversely, the Tuner biomass showed the opposite behavior of TG1, with a yield decrease of 0.004 and 0.029 for 8.0 and 16.0 g/L of AI. However, the decrease observed for 20 g/L of AI was more than double that observed for TG1 (0.069). For TG1, the values obtained are explained in part by the similar final OD at the end of the fermentation study and by the lower xylose consumption (Table 2) in the presence of AI when compared to the control assay. In the case of the Tuner strain, the final OD was lower than the control assay, and likely TG1; xylose consumption was also diminished, generating lower values than the control. Statistically significant effects of the interactions between the “Strain” factor and the “AI” factor were observed on Y_X/S_ (Table 2). Indeed, while the values for Tuner consistently decreased with the increasing AI concentration, those for TG1 increased from 0 to 16 g/L. Notably, for both strains, at 16 and 20 g/L of AI, the values of Y_X/S_ were nearly identical (0.042 and 0.002, respectively) (Table 2). It is noted that the Tuner strain was more affected by AI than TG1.

On the other hand, a weak interaction was observed between the “Strain” and “FI” factors on Y_X/S_ (Table 3). Indeed, while for Tuner, the values of Y_X/S_ did not significantly change from the control to the maximum FI concentration (remaining at an average value of 0.069), the values for TG1 increased with the rising FI concentration (from a minimum of 0.031 to a maximum of 0.047), with the values at 1.5 and 3.0 g/L being nearly identical and equal to 0.038.

The high biomass yields were due to the TG1 strain’s lower consumption of sugar. It was also observed that the TG1 strain had the ability to metabolize part of the acetic and formic acid inhibitors, affecting biomass yield.

The data reported about the consumption of sugars by *E. coli* showed that the bacteria consume xylose with high biomass yields. However, some low yields reported here may be due to the microorganisms used (*E. coli* TG1 e Tuner) consuming a large part of the xylose present in the SMM media [54] and vice versa. It is noted that the time intervals and the concentrations that provide greater biomass production are the same as with the highest sugar consumption, since xylose metabolism is linked to sugar catabolism [54]. Although Zaldivar et al. [57] reported that aeration does not reduce the toxicity of acids, other authors have observed that small amounts of oxygen increase cell growth, promote sugar consumption, and improve redox balance during the fermentation process [58]. As the process was conducted in an anaerobic environment, the absence of oxygen can help to explain the lower growth rates and lower consumption of xylose, even in the presence of inhibitors.

### 3.4. Generation of Products

The sugars present in lignocellulosic materials can be fermented with the aid of microorganisms [59] and converted into several value-added products with applications in the food, cosmetic, and pharmaceutical industries [60]. The Y_P/S_ of acetic acid decreased in both strains but at different rates as the AI concentration increased. Notably, no statistically significant difference was observed in the Y_P/S_ of the acetic acid values for TG1 (averaging 0.31) (Table 2). In contrast, Tuner exhibited more pronounced differences, with values decreasing from 0.324 in the control to a mean of 0.097 at the concentrations of 16 and 20 g/L, with an intermediate value of 0.178 at the lower concentration of AI (Table 2). TG1 also demonstrated superior performance compared to Tuner in the Y_P/S_ of formic acid. A different response was observed between the two strains—TG1 exhibited statistically similar Y_P/S_ formic acid values up to 16 g/L of AI (averaging 0.32) and showed a decrease (0.21) only at the maximum AI concentration (Table 3). In contrast, Tuner exhibited a linear and significant decline in Y_P/S_ of formic acid values, approaching nearly zero as the AI concentrations increased (Table 2). A strong interaction between the factors was observed for the Y_P/S_ of ethanol. Specifically, while TG1 exhibited values that remained constant with varying AI concentrations (averaging 0.082), Tuner experienced a decrease in its Y_P/S_ of ethanol from 0.107 to 0 as the concentration of the inhibitor increased (Table 2). With Tuner, an increase in the Y_P/S_ of lactic acid was observed as the AI concentration increased (from 0.018 to 0.109); however, at the maximum concentration of the inhibitor, the Y_P/S_ was equal to zero (Table 2). In contrast, TG1 did not produce LA at any concentration of AI (Table 2), due to the deletion of lac and proAB genes (Δ(lac-proAB) used for selection in a lactose medium in molecular cloning and gene expression.

In the presence of FI, the Y _P/S_ acetic acid values of the two strains diverged at the low and intermediate concentrations. In particular, the gap between TG1 and Tuner was greatest with FI at 8 g/L (0.39 vs. 0.25) (Table 3). The main factors had highly significant effects on the Y_P/S_ of formic acid; the two strains exhibited the same decreasing trend as the concentration of FI increased, but TG1 consistently displayed higher values than Tuner (on average 0.26 vs. 0.17) (Table 3). The Y_P/S_ ethanol values were divergent, as those for Tuner did not significantly decrease with increasing FI concentrations (averaging 0.108), while TG1 showed a clear decreasing trend (from 0.083 to 0.035) (Table 3). Only Tuner exhibited Y_P/S_ lactic acid, which increased with rising FI concentrations (from 0.018 to 0.060). In contrast, TG1 did not produce lactic acid at all (Table 3).

The production of acetic acid and formic acid followed the same pattern, with production taking place from 3 h to 72 h and eventually reaching similar productions, while ethanol only began to be produced in 9 h but also maintained its production until the end of the fermentation process. Formic acid was produced throughout the fermentation process. Although acetic acid started to be produced in 3 h, it had a higher final production. Ethanol only started to be produced from 30 h and lactic acid from 6 h, with low yield.

#### 3.4.1. Studies with Acetic Acid Inhibitor (AI)

Analyzing Table 3, it is possible to see that there are no significant differences in acetic acid production by the TG1 and Tuner strains. The TG1 strain performed better in generating formic acid, while Tuner produced higher yields in ethanol production and lactic acid, even if only in trace amounts. Although the Tuner strain started ethanol production only after 30 h, it had better yield than TG1.

Acetic acid, formic acid, and ethanol were the products resulting from fermentation with the TG1 strain in the presence of AI, as well as in the control treatment (Figure 1). Compared to the control treatment, a greater amount of acetic acid was produced in the AI concentration of 8.0 g/L (Table 2); no significant differences were observed in the presence of 16.0 g/L AI, while with 20 g/L, a decrease in the yield was observed.

Regarding the formic acid, its production is delayed over time as the concentration of AI increases when compared to the control assay. For 8.0, 16, and 20 g/L of AI, formic acid starts to be produced at 9, 24, and 48 h, respectively (Figure 1). There is no significant difference in the yield of formic acid at the concentration of 8.0 g/L of AI, while at the concentration of 16.0 g/L, there is an increase of 0.021 in the yield, and with 20.0 g/L of AI, a decrease of 0.093 is observed. The production of formic acid is higher at the concentration of 16.0 g/L of AI and is affected at the concentration of 20.0 g/L.

The equivalent pattern, as observed for formic acid, is repeated for ethanol production, in which high concentrations of AI delay the beginning of production. Ethanol starts to be produced in the control treatment after 9 h, while at concentrations of 8.0, 16.0, and 20.0 g/L of AI, it starts at 24, 32, and 54 h, respectively (Figure 1). The yield of this product is 0.08 in both tests; the same was obtained in the control test (Table 2). In general, compared to the control, despite the presence of AI delaying the generation of some products (formic acid and ethanol), the yield of acetic and formic acid was lower and suffered limitations only with the highest concentration of AI (20 g/L) while, with other AI concentrations, the yield was higher or did not change significantly.

The Tuner strain produced acetic acid, formic acid, ethanol, and lactic acid, as well as in the control treatment for this strain (Figure 2). The yield of acetic acid dropped dramatically in the presence of AI, with yield reductions of 0.146, 0.232, and 0.222 at the concentrations of 8.0, 16.0, and 20.0 g/L, respectively (Table 2). Again, formic acid production was delayed by the increasing inhibitor concentration. For 8 and 16 g/L of AI, formic acid production began at 6 and 24 h, respectively (Figure 2). With 20.0 g/L of AI, it was not produced. The yield of formic acid at the concentrations of 8.0 and 16.0 g/L of AI decreased by 0.063 and 0.122 (Table 2). Like formic acid, ethanol was not produced at a concentration of 20.0 g/L of AI. In the control test, this product started to be produced at 24 h, while at concentrations of 8.0 and 16.0 g/L, the production only occurred at 6 h (Figure 2). This could be explained by the metabolization of the inhibitors promoting their production earlier than in the control assay. At the lowest concentration of AI, the ethanol yield was not affected. With the concentration of 16.0 g/L of AI, the yield dropped by 0.062 g/L. At the concentration of 20.0 g/L of AI, the production of lactic acid was also not observed. At concentrations of 8.0 and 16.0 g/L of AI, the lactic acid yield was 0.066 and 0.092 g/L, respectively, notably higher than in the control assay. It was observed that the higher the concentration of AI—not exceeding 16.0 g/L—the greater the lactic acid production by the Tuner strain.

For both TG1 and Tuner, in the three concentrations of AI, the strains followed a similar behavior of high acetic acid production throughout the fermentation process (from 0 to 72 h), with higher yields being correlated to the increasing concentrations of AI. This high production of acetic acid may be correlated with cell growth [54]. With the addition of AI, strain TG1 showed higher or equal yields to the control, except for 20 g/L of AI. In contrast, the Tuner strain reduced yields according to the highest concentrations of AI. These low product yields can be explained not only by the presence of inhibitory compounds but also by the fact that xylose was used as a carbon source both for the formation of energy and products and for cell growth [55].

In the highest concentration of AI, Tuner only produced acetic acid and, even so, with lower yields than in the control. Higher yields for the Tuner strain were only observed in the lactic acid yield at lower concentrations of AI.

#### 3.4.2. Studies with Formic Acid Inhibitor (FI)

During the fermentation process with the TG1 strain in the presence of FI, acetic acid, formic acid, and ethanol were produced (Figure 3), as well as in the control and AI tests. In all the assays, with different FI concentrations (1.5, 3.0, and 6.0 g/L), acetic acid started to be produced from 6 h and persisted until the end of the fermentation process; however, the higher the FI concentration, the lower the production rates. On the other hand, the acetic acid yield at the FI concentration of 1.5 g/L was 0.066 g/L higher than in the control assay. There were no significant differences in the other concentrations (3.0 and 6.0 g/L). It was observed that the acetic acid yield was high at the lowest concentration of FI, but it decreased with increasing concentration (Table 3).

Formic acid was produced during the entire fermentation, from 0 to 72 h, for all the concentrations tested. Furthermore, the higher the concentration of FI, the greater the generation of this product (Figure 3). However, the yield of this product decreased as the inhibitor concentration increased (Table 3).

Zaldivar and Ingram [23] reported that higher concentrations of organic acids progressively inhibited ethanol production. This fact is also proven in the present work, as it was observed that the higher the FI concentration, the slower and smaller the proportion of ethanol generated. For TG1 at the FI concentrations of 1.5, 3.0, and 6.0 g/L, ethanol was produced in 6, 24, and 48 h, respectively (Figure 3). Ethanol yield decreased progressively as the FI increased (Table 3).

The products resulting from the fermentations with the Tuner strain in the presence of FI were acetic acid, formic acid, ethanol, and lactic acid (Figure 4). Acetic acid started to be produced from 6 h, with 1.5 and 3.0 g/L of FI. With 6.0 g/L, the production was from 9 h. Acetic acid yield was smaller than the control assay, and the yield in the presence of FI did not differ significantly between them (Table 3). As for formic acid production, the behavior for Tuner was similar to that of TG1 in terms of the production time. Contrary to TG1, Tuner, at the smallest FI concentration (1.5 g/L), presented a similar value as the control assay. Still, as the FI concentration increased, formic acid yield decreased to values smaller than half of the control assay yield. Ethanol yield in the presence of FI suffered a slight increase in the presence of 1.5 g/L; for higher concentrations of FI, however, ethanol yield did not change (Table 3).

In comparison to the control, higher concentrations of FI led to a greater production of lactic acid. As shown in Table 3, the lactic acid yield increased by 0.012, 0.021, and 0.042 g/L, respectively, when compared to the control assay for the FI concentrations of 1.5, 3.0, and 6.0 g/L. The presence of FI resulted in reduced yields of formic acid production in both strains. The TG1 strain exhibited higher yields of acetic acid but a decrease in ethanol yields. In contrast, the Tuner strain showed higher yields in both ethanol and lactic acid production, along with a decrease in acetic acid production. While FI negatively impacted the production of products primarily for the TG1 strain, this inhibitor aided the metabolism of the Tuner strain, producing greater amounts of valuable products, such as lactic acid and ethanol. It is believed that the pentoses’ metabolism was the major contributor to ethanol production, and the higher ethanol yields by Tuner may be correlated, as reported by Duarte et al. [56]. Presumably, some ethanol was oxidized to biomass and CO_2_ concurrently with the metabolism of the pentoses [61], which may explain the drop in ethanol yield by TG1. The decrease in ethanol production by the presence of the inhibitors for the TG1 strain can potentially result from direct actions in glycolysis and energy generation, in which the ATP produced by substrate-level phosphorylation represents the only energy source for the absorption of nutrients, growth, and homeostasis during fermentation [23]. Parra-Ramírez et al. [37,38,39,40,41,42] reported an ethanol yield of 1.08 g/Lh, while Rios-González et al. [62] obtained a yield of 0.5 g/Lh in media without the addition of inhibitors. Hence, it should be noted that the ethanol yields obtained here did not suffer a potential inhibition by AI and FI.

Many authors have investigated the production of lactic acid by utilizing the sugars found in the hydrolysates of lignocellulosic materials, particularly xylose, which are not metabolized by most microorganisms capable of producing lactic acid [54]. In the literature, *E. coli* is frequently employed for genetic modification and lactic acid production. However, xylose is a pentose that is not metabolized by most lactic acid-producing microorganisms [63]. In the present study, only the *E. coli* Tuner strain was able to produce lactic acid, albeit in small amounts. Parra-Ramírez et al. [54] reported a lactic acid yield of 0.6 g/Lh, while Utrilla et al. [42] had a lactic acid yield of 1.11 g/Lh, both in media without the addition of inhibitors. In the presence of AI, the results obtained in this work showed similar yields to those cited, which shows that AI and FI did not impair the generation metabolism of this value-added product for the Tuner strain.

In a general context, all the results obtained for *E. coli* TG1 and Tuner indicate that cell growth, xylose consumption, and the yields of biomass and products were influenced by the metabolic capacities of their original recombinant strains, K-12 and KL21, respectively. The organic acids inhibited the generation of products during the fermentations mainly by inhibiting the growth of TG1 and Tuner, instead of directly inhibiting the central pathways for glycolysis and energy generation. Zaldivar and Ingram [23] reported that the potency of organic acids was directly correlated with the octanol/water partition coefficients, consistent with a hydrophobic site of action. In this sense, the toxicity of lignocellulosic hydrolysates seems to result from the aggregate effects of various compounds and is observed to be related to hydrophobia.

## 4. Conclusions

Compared to the Tuner strain, the TG1 strain exhibits a greater resistance to acetic acid inhibition, maintaining a more stable growth rate, xylose consumption, and production of ethanol, formic acid, and acetic acid. Although the Tuner strain is more efficient under low-inhibitor conditions, it suffers significant metabolic inhibition at 16–20 g/L AI. TG1’s formic and acetic acid metabolism is less affected, while Tuner loses these pathways at high acetic acid concentrations. Under moderate stress, Tuner compensates by increasing lactic acid production, whereas TG1 does not utilize this adaptation. The Tuner strain shows a greater resistance to formic acid stress, sustaining higher levels of growth, xylose consumption, and ethanol production compared to TG1. TG1, while experiencing a more significant decrease in metabolic activity, upholds stable acetic acid production under stress. Both strains experience inhibition in formic acid metabolism; however, TG1 yields a higher output than Tuner. The Tuner strain adapts by increasing lactic acid production, whereas TG1 does not produce lactic acid at all. Overall, Tuner displays greater robustness in the presence of formic acid, sustaining better metabolic performance than TG1. At the industrial level, TG1 is better suited for applications involving high acetic acid levels, as it maintains its metabolic functions and product yields under stress, while Tuner has better resistance at moderate formic acid concentrations. The use of TG1 for value-added compounds such as ethanol or formic acid may help avoid the use of chemicals to eliminate acetic acid. Tuner could be used for lactic acid production, especially in hydrolysates with under moderate concentration.

## Figures and Tables

**Table 1:** Composition of the SelecTEcoli mineral media (SMM) used in this work.

	Component	Formula	mM
	Xylose	C_5_H_10_O_5_	133.21
	Diammonium hydrogen phosphate	(NH_4_)_2_HPO_4_	19.91
	Ammonium dihydrogen phosphate	NH_4_H_2_PO_4_	7.56
	Potassium dihydrogen phosphate	KH_2_PO_4_	28.29
	Dipotassium hydrogen phosphate	K_2_HPO_4_	71.70
	Magnesium sulfate heptahydrate	MgSO_4_·7H_2_O	1.19
	Potassium hydroxide	KOH	0.99
Vitamin solution	Betaine	C_5_H_10_NO_2_	1.32
	Biotin	C_10_H_16_N_2_O_3_S	0.004
	Thiamine	C_12_H_17_N_4_OS	0.019
	Proline	C_5_H_9_NO_2_	1.99
Trace element solution	Ferric chloride hexahydrate	FeCl_3_·6H_2_O	0.004
	Cobalt chloride hexahydrate	CoCl_2_·6H_2_O	0.0007
	Copper chloride dihydrate	CuCl_2_·2H_2_O	0.0001
	Zinc chloride	ZnCl_2_·4H_2_O	0.002
	Sodium molybdate dihydrate	Na_2_MoO_4_	0.0004
	Boric acid	H_3_BO_3_	0.001
	Manganese chloride tetrahydrate	MnCl_2_·4H_2_O	0.22

**Table 2:** Growth kinetic variables and stoichiometric parameters of biomass and product yields of *E. coli* strains TG1 and Tuner in xylose SMM media, as affected by different concentrations of acetic acid as inhibitors. Different letters within each variable indicates significance at *p* < 0.05; ** indicates significance at *p* < 0.01; *** indicate significance at *p* < 0.001. Ctrl = control; NP = not present; Q_xyl_ = xylose consumption rate; Y_X/S_ = biomass yield; Y_P/S_ = product yield.

Variable	Strain (S)	Acetic Acid Inhibitor (AI) Concentration (g/L)				ANOVA	
		0 (Ctrl)	8	16	20		
Specific growth rate (μ, 1/h) ^a^	Tuner	0.174 a	0.160 a	0.051 c	0.013 c	S AI S × AI	** *** ***
	TG1	0.102 b	0.099 b	0.104 b	0.014 c		
Xylose consumption (%)	Tuner	96.4 a	96.3 a	57.3 bc	22.5 d	S AI S × AI	*** *** ***
	TG1	69.8 b	63.8 bc	49.4 c	50.6 c		
Q_xyl_ (g/L h) ^a^	Tuner	0.270 a	0.269 a	0.160 bc	0.063 d	S AI S × AI	*** *** ***
	TG1	0.191 b	0.178 bc	0.138 c	0.141 c		
Y_X/S_	Tuner	0.071 a	0.067 a	0.042 b	0.002 d	S AI S × AI	*** *** ***
	TG1	0.031 c	0.038 bc	0.044 b	0.002 d		
Y_P/S_ acetic acid	Tuner	0.324 a	0.178 b	0.092 b	0.102 b	S AI S × AI	*** *** **
	TG1	0.322 a	0.357 a	0.327 a	0.227 ab		
Y_P/S_ formic acid	Tuner	0.226 bc	0.163 cd	0.104 d	NP	S AI S × AI	*** *** **
	TG1	0.307 ab	0.311 ab	0.328 a	0.214 bc		
Y_P/S_ ethanol	Tuner	0.107 a	0.104 a	0.045 b	NP	S AI S × AI	*** *** ***
	TG1	0.084 a	0.080 a	0.084 a	0.081 a		
Y_P/S_ lactic acid	Tuner	0.018 a	0.084 b	0.110 a	NP	S AI S × AI	*** *** ***
	TG1	NP	NP	NP	NP		

^a^ Measured between 0 and 72 h.

**Table 3:** Growth kinetic variables and stoichiometric parameters of biomass and product yields of *E. coli* strains TG1 and Tuner in xylose SMM media, as affected by different concentrations of formic acid as inhibitor. Different letters within each variable indicate significance at *p* < 0.05. NS = not significant; * indicates significance at *p* < 0.05; ** indicates significance at *p* < 0.01; *** indicates significance at *p* < 0.001. Ctrl = control; NP = not present; Q_xyl_ = xylose consumption rate; Y_X/S_ = biomass yield; Y_P/S_ = product yield.

Variable	Strain (S)	Formic Acid Inhibitor (FI) Concentration (g/L)				ANOVA	
		0 (Ctrl)	1.5	3	6		
Specific growth rate (μ, 1/h) ^a^	Tuner	0.174 a	0.143 ab	0.134 ab	0.114 b	S FI S × FI	*** *** NS
	TG1	0.102 b	0.061 c	0.049 c	0.034 c		
Xylose consumption (%)	Tuner	96.4 a	96.3 a	91.1 a	56.3 bc	S FI S × FI	*** *** **
	TG1	69.8 b	44.4 cd	43.5 cd	32.9 d		
Q_xyl_ (g/L h) ^a^	Tuner	0.270 a	0.270 a	0.256 a	0.158 bc	S FI S × FI	*** *** **
	TG1	0.191 b	0.124 cd	0.122 cd	0.092 d		
Y_X/S_	Tuner	0.071 a	0.067 a	0.065 a	0.072 a	S FI S × FI	*** * *
	TG1	0.031 c	0.038 bc	0.038 bc	0.047 b		
Y_P/S_ acetic acid	Tuner	0.324 ab	0.246 b	0.248 b	0.288 ab	S FI S × FI	*** NS *
	TG1	0.322 ab	0.388 a	0.327 ab	0.359 ab		
Y_P/S_ formic acid	Tuner	0.226 b	0.200 b	0.162 bc	0.092 c	S FI S × FI	*** *** NS
	TG1	0.307 a	0.303 a	0.219 b	0.201 b		
Y_P/S_ ethanol	Tuner	0.107 ab	0.118 a	0.107 ab	0.101 ab	S FI S × FI	*** *** **
	TG1	0.084 bc	0.067 cd	0.048 de	0.035 e		
Y_P/S_ lactic acid	Tuner	0.018 c	0.030 bc	0.039 b	0.060 a	S FI S × FI	*** ** **
	TG1	NP	NP	NP	NP		

^a^—Measured between 0 and 72 h.

## Data Availability

The original contributions presented in this study are included in the article. Further inquiries can be directed to the corresponding author.
